# Expanding mammography screening for women aged 40–80 years: evidence from a modeling approach using real-world data

**DOI:** 10.1038/s41598-023-42820-9

**Published:** 2023-09-27

**Authors:** Dieter Hölzel, Kathrin Halfter, Gabriele Schubert-Fritschle, Jutta Engel

**Affiliations:** Institute of Medical Information Processing, Biometry and Epidemiology (IBE), Faculty of Medicine, Ludwig-Maximilians University Munich, Marchioninistraße 15, 81377 Munich, Germany

**Keywords:** Epidemiology, Breast cancer, Cancer screening

## Abstract

If a mammography screening program (MS) is to be expanded, the benefit must be demonstrated for each additional age cohort. For the age interval between 40 and 80 years, the association between tumor-related and tumor-independent mortality of 21 2-year cohorts is modeled using up-to-date, valid data to determine MS outcome. Disease trajectories with and without biennial MS are extrapolated for each age cohort using the available data and knowledge on MS. The competing mortality is randomly generated for each age cohort with and without MS for a follow-up period of 20 years. Analyses of the modeled cohorts describe incremental change for each year, quantifying the changing benefits of MS. With increasing age, the proportion of tumor-independent mortality before and with metastatic disease increases and the benefit decreases. The simulations with 21 studies on the age interval 40–80 years provide four parameters to determine the benefits and costs of MS: The number of prevented deaths, required mammography screening exams (MSE) and their costs, life-years gained, and the required MSEs. If one additional MSE is offered for age groups 48/70 years, this will result in 311/320 prevented breast cancer (BC) deaths with 1742/1494 required MSEs or 8784/4168 life-years gained with 64/140 required MSEs. A rational cutoff cannot be quantified. The mortality effect of MS between 40 and 80 years is quantified in 21 steps using two metrics, number of MSEs per tumor-related mortality prevented and per life-year gained. This provides a decision support for stepwise expansions. Given this real-world evidence no rational age cutoffs for MS becomes evident. A society has to decide which MS costs, including side effects of MS for women who remain BC-free, it is willing and able to accept in order to reduce breast cancer mortality.

## Introduction

The subject of whether or not mammography screening programs (MS) should be expanded to include additional age groups needs to be reassessed continuously^1–3^. That is because more recent data and research may lead to updated conclusions and recommendations. For example, in women aged 70 years and older, there is only one ongoing randomized trial studying the effectiveness of MS to date^4^. In order to update current recommendations there are many findings to consider, like the already available results from clinical trials and public health institutions, observations on long disease courses or tumor independent mortality, all of which can be used to model MS outcome. Based on the 2021 edition of the *Mammo REPORT* published by the Kooperationsgemeinschaft Mammographie, a total of 13,414 invasive breast cancers (BCs) were diagnosed out of the 2.92 million mammography screenings which were conducted in 2018. The Kooperationsgemeinschaft Mammographie is a German collaborative which collects data from five regional reference sites. It is financed by health insurance providers and aims to ensure high quality care, collects and analyzes data, and provides guidance on mammography screening^5^. Using these and other available data, real-world evidence on MS may be obtained where feasibility and time constraints complicate the design of a randomized controlled trial following a similar objective. Data from various sources are required for any modeling approach to ensure validity and representative results. The aim of this work is to apply a modeling approach to age-specific screening cohorts in order to describe the dynamics of the interaction between tumor-dependent and tumor-independent mortality. Based on these results new or additional criteria for modifications of the MS guidelines may be considered.

## Methods

The modeling of MS described below is based on the age at the initial MS examination (MSE) beginning at 40 up until and including 80 for each of two cohorts (Supplementary Table S1). The size of the screening cohorts corresponds to the current population structure as published by the Federal Statistical Office of Germany (Supplementary Table S1-Population)^6^. The summary of the screening cohorts shown in table S1 of the supplement describes the results of the modeled biennial screening in Germany.

The probability “to survive another year” is estimated based on the expected survival of the general population taken from the official mortality tables^7^. In Supplementary Table S1-life expectancy, the decreasing number of expected life years with age is given, resulting in the loss of life years from tumor-related death. The BC incidence from current data for Germany provided by the Robert-Koch-Institut (RKI) and survival data from the Munich Cancer Registry (MCR), a cancer registry with data from a catchment area of currently 5.16 million inhabitants (Supplementary Table S1-Incidence) are used for modeling. Due to the higher BC incidence in elderly women in the United States, screening effects are also estimated on the basis of SEER data (Supplementary Table S5)^8, 9^. According to the given incidence, the number of estimated newly diagnosed cases in the population in 1 year is reported for each cohort (Supplementary Table S1-BC). Only 80% of these will be diagnosed by regular MSE within MS, the other cases are diagnosed as unavoidable interval BCs or outside of MS (Supplementary Table S1-BC).

Tumor size is a main prognostic factor in BC and the tumor diameter (TD) is used for modeling. Using the MCR survival data on TD in 5-mm increments, a Gompertz function was fitted for the relationship between TD and 15-year survival^10^.

$$15yr\,tumor - related\,survival = 100 - 58.4e^{{\left( { - 4.46e^{{\left( { - 0.071*TD} \right)}} } \right)}}$$

The hazard rates for modeling survival are specified per TD-specific survival.

**Figure 1:**
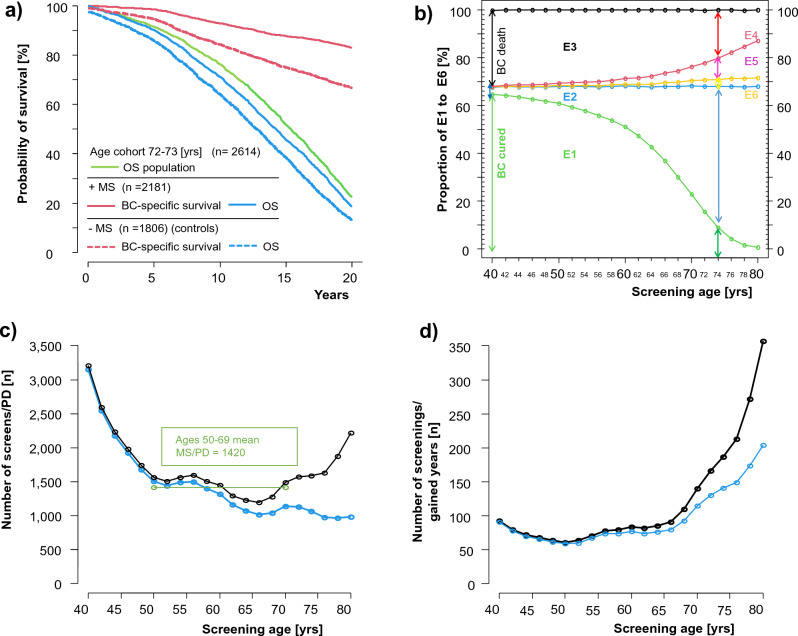
Results of MS modeling. (**a**) Simulation results for the age cohorts 72–73 years and 20-year survival of BCs detected with (+ MS) and without screening (− MS); (**b**) probability distribution of the endpoints E1–E6 by age in the 21 cohorts without screening. Endpoints are defined as follows: E1 = no event occurs during 20 years of follow-up, E2 = occurrence of tumor-independent deaths only, E3 = tumor-dependent deaths are always expected but in part prevented by tumor-independent death as the first event, E4 = tumor-related death is the first event to occur, E5 = tumor-independent death is the first event to occur prior to metastasis, E6 = tumor independent death occurs following diagnosis of metachronous metastasis. (E1 + E2 + E3 = 100%, E3 = E4 + E5 + E6). (The small proportion of BC-related deaths after 20 years is not shown in the figure or in the supplementary tables). (**c**) Age-dependence of the number of screens to prevent one tumor-related death compared to the − MS cohort (blue: with/black without corrections for tumor-independent mortality as reported in supplementary Tables S2/S3. (**d**) The number of screens needed to gain one year of life according the age at screening.

The reported tumor size distribution based on data from MS published by *Mammo REPORT* (≤ 10 mm/> 10-20 mm/> 20 mm) was 33.8%/46.0%/18.7% respectively^5^. If a mean of 7 mm/15 mm/28 mm in TD for the three classes is assumed, the overall weighted mean TD is 14.7 mm. According to the Gompertz function above, this results in a 15-year survival of 87.8%. Considering interval cancers, we assumed 16 mm or 86% survival and extrapolated to 81.9% for 20 years (Table 1). These numbers are used in the modeling analysis with MS (+ MS) (Fig. 1a). For the mean tumor size without MS (− MS), the 15-year survival is assumed to be 76%, which according to the Gompertz function comes to 67.9% after 20 years for a BC with a TD of 22.7 mm.

**Table 1:** Modeled age specific populations (MP) of the female population in Germany and 20 years survival with and without MS.

	Simulated 20-yr survival after BC diagnosis (%)				
	OS Population	+ MS		− MS	
		BC-specific	OS	BC-specific	OS
Modeled populations					
MP 40	94.5	80.6	77.3	66.2	62.1
MP 48	91.3	83.9	77.0	67.4	61.5
MP 50	88.6	82.2	72.5	67.7	59.8
MP 60	75.2	81.9	60.3	68.9	52.2
MP 70	33.4	81.9	26.4	68.4	22.9
MP 72	22.6	83.4	19.5	69.1	15.2
MP 80	0.6	81.2	0.7	68.7	0.4
Sum/weighted means					
SI 50–69	72.3	81.9	59.2	67.8	48.9
SI 40–80	57.8	81.9	47.5	67.9	39.1

Full list of MPs shown in supplementary table.

*BC* breast cancer cases, *M* Million, *MS* mammography screening program, *OS* overall survival, *SI* Age interval for screening.

The disease trajectories for the cohorts MS+/MS− are derived from the two survival functions through which tumor-related death is generated. The same tumor-related mortality is assumed for each screening age, because according to our data the proportion of HR− cancers doubles only below 40 years of age. In addition, the prognosis of HR + BCs actually worsens after 15 years. The total cost of the MS program in Germany is approximately 88€ per screening which are covered by the health insurance provider (Kooperationsgemeinschaft Mammographie, e-mail communication, July 2023).

### Modeling of the screening

Using the current findings from the German MS and estimated 20-year survival, the two events tumor-dependent and tumor-independent death are randomly generated during 20 years of follow-up. Disease trajectories for screening participants and non-participants are created for each of the 21 modeled populations. For any given screening age, the disease trajectories expected in the MS+/MS−cohorts are reported. Stepwise, for each of the 20 years of follow-up, the year of death is estimated according to the hazard rates based on the life table or on the survival function. The date of death within the year is estimated following an equal distribution.

In total, 21 cohorts with and without MS are modeled for ages 40–80 years at 2-year intervals. Both of these modeled cohorts correspond to randomized trials and are assessed for all ages.

The random generation of the events results three different endpoint groups (E): E1 and E2 are cured BC patients, E3 are expected BC-related deaths.

The unique feature of this modeling approach is the combination of the two events with the event of metastasis (MET) about 28 months before BC-related death. This yields three additional patient-relevant endpoints for disease progression, namely E4-E6 (Fig. 1b, Tables 2, 3).

**Table 2:** Distribution of the six patient-relevant endpoints E1 to E6 in the modeled screening cohorts after 20 years of follow-up. Endpoints E1–E6 see text.

	E1 (%)	E2 (%)	E3 (%)	E4 (%)	E5 (%)	E6 (%)	LL	LL corr.^a^	YLL	YLL corr.^a^
Modeled screening cohort										
MS 40	78.50	3.45	18.05	17.77	0.21	0.07	207	204	6900	6750
MS 48	74.73	7.20	18.07	17.43	0.46	0.18	411	397	10,740	10,309
MS 50	73.19	8.70	18.11	17.28	0.62	0.21	527	503	12,825	12,191
MS 60	61.34	20.38	18.28	16.36	1.44	0.48	641	574	10,321	9286
MS 70	27.24	54.65	18.10	13.24	3.59	1.28	554	405	4966	3884
MS 72	18.54	63.36	18.10	12.22	4.36	1.51	473	320	3712	2759
MS 80	0.60	81.13	18.27	6.79	9.39	2.09	616	229	2632	1283
Sum/weighted means										
SI 50–69	59.20	22.70	18.20	16.10	1.50	0.50	6158	5462	103,557	93,911

*LL* lives lost, *SI* Age interval for screening, *YLL* years of life lost.

^a^Adjusted for competing events E5 or E6.

**Table 3:** Distribution of the patient-relevant endpoints E1 to E6 in the modeled control population cohorts without screening after 20 years of follow-up. Endpoints E1–E6 see text.

	E1 (%)	E2 (%)	E3 (%)	E4 (%)	E5 (%)	E6 (%)	LL	LL corr.^a^	YLL	YLL corr.^a^
Modeled control cohort										
MC 40	64.74	2.91	32.35	31.86	0.35	0.14	371	365	12,578	12,318
MC 48	61.66	6.10	32.24	31.13	0.82	0.29	733	708	19,524	18,780
MC 50	60.86	7.23	31.90	30.62	0.95	0.34	928	890	23,079	22,062
MC 60	51.18	17.02	31.79	28.67	2.34	0.78	1116	1006	18,475	16,780
MC 70	22.87	45.31	31.82	23.70	5.86	2.25	973	725	9134	7291
MC 72	15.42	52.64	31.94	22.13	7.23	2.57	835	579	6845	5220
MC 80	0.47	67.46	32.07	12.90	15.54	3.63	1081	435	4870	2562
Sum/weighted means										
SI 50–69	49.10	18.80	32.10	28.70	2.50	0.90	10,875	9738	188,139	172,128

*LL* lives lost, *SI* age interval for screening, *YLL* years of life lost.

^a^Adjusted for competing events E5 or E6.

The description of the endpoints groups is as follows:

(A) Events within 20 years of follow-up:
  *E1* No event occurs during 20 years of follow-up.
  *E2* Only tumor-independent deaths occur.
  *E3* Tumor-dependent deaths occur and in part prevented by tumor-independent deaths.
(B) Patient-related endpoints depending on the sequence of events and in relation to MET:
  *E4* Tumor-related death is the first event.
  *E5* Tumor-independent death is the first event, MET were not diagnosed.
  *E6* Tumor-independent death occurs after MET has already been diagnosed.

MS should reduce the number of years of life lost (Tables 2, 3-YLL). These effects of MS are obtained by comparing the cohorts with and without MS.

Each simulation is repeated 50 times to reduce random generation variability and the resulting mean values are reported. Using the differences in the characteristics of the modeled cohorts, the incremental changes for each age level are determined. Results are tabulated and graphed for each of the 21 age groups starting from 40 to 80 years of age. The modeling and statistical analyses are conducted using the R system version 3.1.3^11^.

## Results

The applied assumptions and their respective results for all of the modeled cohorts are provided in Tables 1, 2, 3 and 4 with relevant excerpts and in full detail for the 21 age groups in the Supplementary Tables S1–S4. BC-specific and overall survival depends on age as exemplified for MC72 in Fig. 1a, which models the expected 20-year survival of the running AgeX study^4^.

**Table 4:** Screening effects comparing modeled cohorts with (+ MS) and without screening (− MS) after 20 years of follow-up. Cost per screening examination € 88.

	PD	PD corr.^a^	MSE per PD corr.^a^	€ per PD corr.^a^	LY corr.^a^	MSE per LY corr.^a^	€ per LY corr.^a^	PD per 10^4^ WS	LY per 10^4^WS
∆ + MS/− MS (Tables 2, 3)									
∆40	164	161	3208	282,000	5678	93	8200	3.1	108
∆48	323	311	1742	153,000	8784	64	5600	5.7	156
∆50	401	387	1566	138,000	10,253	61	5400	6.4	163
∆60	475	432	1450	128,000	8154	84	7400	6.9	120
∆70	419	320	1494	131,000	4168	140	12,300	6.7	71
∆72	362	259	1577	139,000	3133	166	14,600	6.3	60
∆80	465	206	2218	195,000	2238	357	31,400	4.5	28
Sum/weighted means for 10 Studies									
SI 50–69	4718	4276	1413	124,000	8387	81	7200	7.1	126

*LY* life years gained, *MS* mammography screening, *PD* prevented deaths, *S* screening examinations, *SI* age interval for screening, *WS* women screened.

^a^Adjusted for competing events.

From the incidence, number of women in the 2-year interval, and 20-year survival of 81.9%/67.9% with and without MS the expected and preventable BC deaths are calculated (Table 4). Expected mortality reduces this effect, for which three endpoints must be considered:

1. *E4* Tumor-related death is the first event. At a younger age tumor-independent death rarely occurs during 20 years follow-up, with MS for MC40/MC72 in 3.5/63,4%.
2. *E5* Tumor-independent death is the first event, MET does not occur in these BC patients. Due to the long progression-free phase in BC before late MET, the proportion of patients in this group increases with age, with MS for MC40/MC72 in 0.2/4.4%.
3. *E6* Tumor-independent death occurs after metastatic disease has already been diagnosed and also increases with age, with MS for MC40/MC72 in 0.07/1.51%. E5 corresponds to triple the value of E6 and states that the metastasis-free time is 84 months or 7 years on average. This dynamic of the endpoints as a function of age is described in Fig. 1b.

In Table 4 the differences in tumor-related deaths and life-years lost from Tables 2, 3 are compiled and represent deaths prevented (PD+/− competing events) and life-years gained (LY).

The distribution of PD adjusted for competing events (PD corr.) follows the incidence and is concave. The reference to the number of MSE repeated every 2 years is thus convex (Fig. 1c). For MC40 it follows from the low incidence and for MC80 from the high normal mortality that 3208/2218 MSE are required for one PD corr. An optimum (minimum MSE) is achieved at MC66 with 1199 MSE.

Incidence and life expectancy and their difference in the time to metastasis or tumor-related death result in the years of life lost (YLL). Their difference with and without MS are the life years gained according to the required MSE (Table 4-LY,S/LY), which are shown in Fig. 1d.

The benefits of MS are easier to communicate if the results are based on 1000 women leading to 10,000 MSE in 20 years by a 2-year MS (Table 4 PD corr. or LY per 10^4^ WS). In the age interval recommended for screening (SI) of 50–69 years, 7.1 BC deaths are prevented and an estimated 57 BC are diagnosed in the subsequent 20 years follow-up. Most patients will undergo breast-conserving surgery, nevertheless about 10 women will die of BC despite MS.

The overall cost of the MS is calculated by multiplying the cost of MSE, in Germany 88€ per MSE with MSE per PD corr. Thus, they can be compared with the treatment costs of BCs, the total primary therapy to prevent BC death, and the progression therapy to prolong survival.

In the summary row, the modeling results for all 12.1 million women eligible for MS are shown. If all these women participated in this screening program a reduction of 4276 BC cases with 1413 MS for one PD corr. could be achieved.

If an 11th MSE is offered, starting at MC48 or expanding to include MC70, at least four aspects from Table 4 have to be considered: (1) For MC48/MC70 311/320 BC-deaths can be prevented; (2) To prevent one death 1742/1494 MSE are required or with 88€/MSE almost 22,000€ more are necessary in MC48; (3) Furthermore, the number of years of life gained (8784/4168 LY), based on a life expectancy of 37.2/17.7 years; (4) 64/140 MSE are required for 1 year of life gained, which corresponds to 5623/12,320€ (according to cost figures provided by the Kooperationsgemeinschaft Mammographie, e-mail communication, Juli 2023).

However, these parameters do not suggest a rational decision on a potential cutoff.

These simulations are also compiled with the SEER incidence for the US population in the supplementary data (Supplementary Table S5). The higher incidence from MC54 reduces the number of MSE required and thus increases the benefit.

## Discussion

In this modeling approach, the benefit of MS in the age interval between 40 to 80 years is presented. The modeling approach was performed using valid epidemiological and follow-up data of 20 years. The results described above demonstrate the effect of MS estimated through the modeling of age-specific screening studies. A 20-year survival of approximately 81.9% is expected for all BCs diagnosed within MS. In the absence of MS, a survival of 67.9% is expected which is equivalent to the currently observed population-based BC data^8, 9^. This modeling approach generated age-specific disease trajectories, with five patient-relevant endpoints distinguished at follow-up. With the absence of tipping points or drastic changes in diagnosis, therapy, or disease progression in BC, the modeling data describes the incremental changes between 40 and 80 years of age. These results vary from previously published approaches which estimated global ratios for varying SI^12–14^ and therefore present a new approach in modeling the effects of MS.

An important factor for the MS effect is the difference in 20-year survival with or without MS, which comes to 81.9–67.8% according to our results in the SI of 50–69 years. In Germany, there were 18,519 BC deaths in 2019 with 50% MS participation^15^. Without MS 20,657 and with 100% MS 16,381 BC-death would be expected, a reduction of 20.7%. This reproduces previously published studies and meta-analyses^16, 17^.

If, for example, this difference is 1% greater, 7.2% or an estimated 30 additional BC deaths would be prevented by MS. For MC60, one death would be avoided with 1350 instead of 1450 MSE, and 10,000 MSE would prevent 7.4 instead of 6.9 deaths. Effectiveness would decrease if the difference is smaller. This could go as far as a reassessment of a screening program if adjuvant therapy for BC were decisively improved. The second factor is the incidence within an age group. The corresponding simulation with the higher SEER incidence from MC54 and the US population is shown in Table S5 of the supplementary material.

The validity of the modeling approach can be illustrated by comparison to the data of the UK Age trial^18^. The supplementary data shows almost identical numbers of BCs in the intervention and control group for 40-year-olds after translation to person years and 10 years of follow-up.

Within the existing SI (50–69 years) the results above are a comparable interpolation of published and available MS-results. All age cohorts outside of this recommended SI are extrapolated and need to be validated in the future by results of 20 year follow-up within ongoing trials. Whether this simulation will correctly estimate the 20-year outcome of the AgeX trial or other current estimates remains to be seen^19^. In particular, determining the benefit in younger women is very challenging since 20 years of follow-up is required. An expansion to ages above or below 50–69 requires a higher number of MSEs to prevent one BC death or gain one additional year of life (Fig. 1c, d).

In our opinion, similar results have not been shown in other studies to date, which may overestimate the benefit of MS due to the lack of differentiated endpoints. With a reduction of age-related BC events due to competing life expectancy, MS appears equally effective for ages 48/70 in terms of the number of mammograms for a year of life gained (Fig. 1c, Table 4-MSE/PD corr.). Therefore, a second aspect, the number of mammograms for a year of life gained should be considered. Here, due to the difference in life expectancy (37.2/17.7 years) there is a large effect in 8784/4168 LY adjusted for competing events, these numbers favor the MC48.

Figure 1c, d illustrates that two aspects have to be considered for the entire age interval 40–80 years. To prevent BC death 3208 MSE are required in MC40. The lowest number of MSE are required in MC66 (n = 1199). The numbers continue to increase with 2218 required in MC80. However, this increase is not due to a rising incidence, rather this reduction in BC mortality is affected by competing expected mortality. In the MC40 this reduction only comes to 161/164 (98.2%) and decreases to 206/465 (44.3%) in the MC80. The resulting distribution is u-shaped and therefore cannot offer a cut-off point based on rational arguments.

In contrast, the number of MSE to gain 1 year of life increases steadily with the exception for women in their 40 s where an incidence effect becomes apparent. In the MC80 a total of 10,000 MSE are required to gain 28 years of life.

This ultimately leads to the question how a society values the same number of years of life gained by a smaller number of young patients compared to those gained by many older patients (Table 4: MC50/72 years with 61/166 MSE/LY corr. Fig. 1d).

The idea of validating the benefit of MS for further age intervals with studies is not feasible. Our available knowledge is so evident that no woman can be offered participation in a control group and 10 or more years of follow-up.

Costs shown in Table 4 are intended to illustrate the contrast between the costs of the preventive measure on an individual case basis (10 biennial MSE in 20 years). In women ages 50–69 years a total of 1413 S or, about 125,000 € (at 88€ per S * 1413) are required to prevent one death. These costs could be compared to those of primary and adjuvant therapy. The cost of one life year gained are 81 MSE or 7128€. Both these factors could be assessed in relation to progression therapies.

In circumstances where public health insurance is available, the costs reflect what an organized MS is worth to a community. In Germany, the participation rate in MS remains constant at 50%, an increase in participation has the potential to avoid more than 2000 BC-related deaths. Achieving a participation rate of 70% and more with better information on outcomes for eligible women is a guideline requirement and remains a constant issue for all involved parties. If an effort to increase the patient participation rate remains unsuccessful, the question arises whether there is a limit below which no MS may be offered. Services with low patient adherence are not sustainable in a health care system and may be discontinued.

In general, modeling studies such as this one provides estimates and generates hypothesis, however clinical studies remain essential in evidence-based decision-making processes. A randomized trial and missing tipping points are prerequisites for any modeling approach. Only if this is given can modeling provide relevant results within and outside the given SI of the available study data.

Only mortality effects were considered in the modeling approach described above. The previously reported and well-known association between tumor size, mastectomies, over diagnosis^20^, the age-dependence of false positive findings due to breast density, and the clarification process of suspicious findings, are patient-relevant aspects that may influence quality of life and are not included in the model. These also include less burdensome treatment options that are available for early tumor stages. The burden for women who remain free of findings in 10 or more screenings, the recall rate, or even the DCIS rate are also aspects that should be especially considered in age-specific recommendations. Age-specific data on such effects can be included in the modeling and may provide another aspect in the assessment of MS.

Endpoints such as number of deaths avoided or years of life gained are ratios that can be used to compare the benefit of MS at 48 and 70 years. This quantification allows for comparisons between ages and thus for reasoned and fair medical decisions regarding screening expansions. However, there is no decision criterion that clinical studies avoid through their yes–no decisions.

## Conclusions

With the results presented here, the discussed extension of the age limits of screening can be assessed. For MC48/MC70, 5.7/6.7 BC deaths can be prevented relative to 10^4^ MSE, but with a significant difference in life years gained from 156/71 to 10^4^ MSE. Therefore, with each offer for an additional MS examination, a society and its public health institutions must reflect on the adequate costs of a preventable death that can be achieved with the best and safest method today to reduce BC mortality. A similar recommendation based in part on the above presented data was recently published by the German agency responsible for assessing the quality of screening methods (IQWiG)^3^.

### Supplementary Information

Supplementary Information.

## Data Availability

The human data supporting the conclusions of this article are publicly available in the following repositories: Population data: Statistisches Bundesamt. Bevölkerung und Erwerbstätigkeit Fachserie 1 Reihe 1.3. 2020. (https://www.destatis.de/DE/Themen/Gesellschaft-Umwelt/Bevoelkerung/Bevoelkerungsstand/Publikationen/Downloads-Bevoelkerungsstand/bevoelkerungsfortschreibung-2010130207005.html). Mortality tables: Statistisches Bundesamt. Sterbetafel 2015/17. 2018. (https://www.statistischebibliothek.de/mir/receive/DEHeft_mods_00096671). Breast cancer incidence and survival from the Munich Cancer Registry (http://www.tumorregister-muenchen.de/facts/specific_analysis.php).

## Abbreviations

BC: Breast cancer
E: Endpoint group
HR: Hormone receptor status
LY: Life years gained
MC: Modeled cohort
MCR: Munich Cancer Registry
MET: Metastasis
MS: Mammography screening program
MSE: Mammography screening examination
PD: Prevented death
PD corr: Prevented death adjusted for competing events
S/LY: Screenings per life year gained
SI: Age interval for screening
TD: Tumor diameter
WS: Women screened
YLL: Years of life lost

## References

[1] Canelo-Aybar C, Ferreira DS, Ballesteros M (2021). Benefits and harms of breast cancer mammography screening for women at average risk of breast cancer: A systematic review for the European Commission Initiative on Breast Cancer. J. Med. Screen..

[2] Schünemann HJ, Lerda D, Quinn C (2020). Breast cancer screening and diagnosis: A synopsis of the European breast guidelines. Ann. Intern. Med..

[3] IQWiG. Überprüfung der Altersgrenzen im Mammografie-Screening-Programm. 2022; s21–01.

[4] University of Oxford. Nationwide cluster-randomised trial of extending the NHS breast screening age range in England (2020). https://www.isrctn.com/ISRCTN33292440.

[5] Mammographie K. Mammoreport Februar 2021: Daten und Fakten zum deutschen Mammographie-Screening-Programm 1–4 (2021).

[6] Statistisches Bundesamt. Bevölkerung und Erwerbstätigkeit Fachserie 1 Reihe 1.3 (2020). https://www.destatis.de/DE/Themen/Gesellschaft-Umwelt/Bevoelkerung/Bevoelkerungsstand/Publikationen/Downloads-Bevoelkerungsstand/bevoelkerungsfortschreibung-2010130207005.xlsx?__blob=publicationFile.

[7] Statistisches Bundesamt. Sterbetafel 2015/17 (2018). https://www.statistischebibliothek.de/mir/servlets/MCRFileNodeServlet/DEHeft_derivate_00042865/5126203177004.pdf.

[8] Tumorregister München. http://www.tumorregister-muenchen.de/facts/specific_analysis.php (aufgerufen Sept. 2021).

[9] Noone, A., Howlader, N., Krapcho, M., Miller, D. & Brest, A. SEER cancer statistics review, 1975–2017 National Cancer Institute. Bethesda, MD. http://seer.cancer.gov/. Accessed 10 Nov 2021.

[10] Engel J, Weichert W, Jung A, Emeny R, Hölzel D (2019). Lymph node infiltration, parallel metastasis and treatment success in breast cancer. Breast (Edinburgh, Scotland).

[11] R Core Team. R: A language and environment for statistical computing. R: Foundation for Statistical Computing, Vienna, Austria (2015). http://www.R-project.org/.

[12] Kregting LM, Sankatsing VDV, Heijnsdijk EAM, de Koning HJ, van Ravesteyn NT (2022). Finding the optimal mammography screening strategy: A cost-effectiveness analysis of 920 modelled strategies. Int. J. Cancer.

[13] Yaffe MJ, Mittmann N, Lee P (2015). Clinical outcomes of modelling mammography screening strategies. Health Rep..

[14] Mühlberger N, Sroczynski G, Gogollari A (2021). Cost effectiveness of breast cancer screening and prevention: A systematic review with a focus on risk-adapted strategies. Eur. J. Health Econ..

[15] Zentrum für Krebsregisterdaten im Robert Koch-Institut: Datenbankabfrage mit Schätzung der Inzidenz, Prävalenz und des Überlebens von Krebs in Deutschland auf Basis der epidemiologischen Landeskrebsregisterdaten (DOI: 10.18444/5.03.01.0005.0017.0001 [Inzidenz). Mortalitätsdaten bereitgestellt vom Statistischen Bundesamt. www.krebsdaten.de/abfrage, Letzte Aktualisierung: 13.09.2022, Abrufdatum: (08.08.2023).

[16] Gotzsche PC, Nielsen M (2011). Screening for breast cancer with mammography. Cochrane Database Syst. Rev..

[17] Tonelli M, Connor Gorber S, Joffres M, Dickinson J, Singh H, Lewin G (2011). Recommendations on screening for breast cancer in average-risk women aged 40–74 years. CMAJ.

[18] Duffy SW, Vulkan D, Cuckle H (2020). Effect of mammographic screening from age 40 years on breast cancer mortality (UK Age trial): Final results of a randomised, controlled trial. Lancet Oncol..

[19] Duffy SW, Tabár L, Yen AM (2021). Beneficial effect of consecutive screening mammography examinations on mortality from breast cancer: A prospective study. Radiology.

[20] Welch HG, Prorok PC, O'Malley AJ, Kramer BS (2016). Breast-cancer tumor size, overdiagnosis, and mammography screening effectiveness. N. Engl. J. Med..

