# Supplementary material for: Expanding mammography screening for women aged 40–80 years: evidence from a modeling approach using real-world data
Source: Sci Rep. 2023 Sep 27;13:16229. doi: 10.1038/s41598-023-42820-9 (PMC10533880; doi:10.1038/s41598-023-42820-9)
Supplement: Supplementary file 1 — Supplementary Information. [file 41598_2023_42820_MOESM1_ESM.docx]

**Supplementary appendix**

is part of the original submission

Supplement to:

*Expanding mammography screening for women aged 40 - 80 years: Evidence from a modeling approach using real world data*

Dieter Hölzel, Kathrin Halfter, Gabriele Schubert-Fritschle, Jutta Engel

**Table S1:** Modeled age specific populations of the female population in Germany and 20 years survival with and without MS. The number of mammography screens depends on the German population structure that is characterized by a decline in births both around 1945 and 1961 after birth control become available (MP74-76 / MP60 +). Data sources for population data and life expectancy is the Federal Statistical Office of Germany, BC incidence and survival are from the database of the Munich Cancer Registry. 20-year survival data after BC diagnosis (Overall, with mammography screening (+MS) or without (-MS) was modeled using the results of the Gomperzt function given in the methods section according to reported tumor diameter, then extrapolated to 20 years follow-up.

|  |  | **Modeling Data** | | | | | **20-yr survival after BC diagnosis [%]** | | | | |
| --- | --- | --- | --- | --- | --- | --- | --- | --- | --- | --- | --- |
|  |  |  |  |  |  |  |  | **+MS** | | **-MS** | |
|  |  | **Age^a^ [yrs]** | **Population**  **[M]** | **Life expectancy [yrs]** | **Incidence per 10^5^** | **BC [n]^b^** | **OS**  **Population** | **BC-**  **specific** | **OS** | **BC-specific** | **OS** |
| **Modeled populations** | MP 40 | 40 | 1,03 | 44.9 | 111.0 | 1147 | 94.5 | 80.6 | 77.3 | 66.2 | 62.1 |
|  | MP 42 | 42 | 0,99 | 43.0 | 141.0 | 1392 | 95.0 | 82.0 | 78.4 | 67.9 | 65.2 |
|  | MP 44 | 44 | 0,95 | 41.0 | 165.0 | 1572 | 94.1 | 82.5 | 77.2 | 65.8 | 62.0 |
|  | MP 46 | 46 | 0,96 | 39.1 | 186.0 | 1780 | 91.7 | 80.8 | 74.3 | 68.5 | 62.5 |
|  | MP 48 | 48 | 1,08 | 37.2 | 210.0 | 2275 | 91.3 | 83.9 | 77.0 | 67.4 | 61.5 |
|  | MP 50 | 50 | 1,21 | 35.3 | 240.0 | 2909 | 88.6 | 82.2 | 72.5 | 67.7 | 59.8 |
|  | MP 52 | 52 | 1,32 | 33.4 | 250.0 | 3311 | 86.5 | 82.5 | 72.5 | 67.1 | 57.9 |
|  | MP 54 | 54 | 1,37 | 31.6 | 240.0 | 3292 | 83.7 | 82.1 | 69.4 | 67.0 | 56.0 |
|  | MP 56 | 56 | 1,39 | 29.7 | 235.0 | 3263 | 82.4 | 81.4 | 67.6 | 67.5 | 55.7 |
|  | MP 58 | 58 | 1,33 | 27.9 | 255.0 | 3388 | 79.5 | 82.0 | 64.6 | 67.5 | 53.8 |
|  | MP 60 | 60 | 1,25 | 26.2 | 280.0 | 3509 | 75.2 | 81.9 | 60.3 | 68.9 | 52.2 |
|  | MP 62 | 62 | 1,15 | 24.4 | 310.0 | 3558 | 70.2 | 81.5 | 57.1 | 68.0 | 47.3 |
|  | MP 64 | 64 | 1,09 | 22.7 | 330.0 | 3581 | 63.1 | 81.3 | 52.1 | 68.0 | 42.7 |
|  | MP 66 | 66 | 1,03 | 21.0 | 360.0 | 3721 | 54.1 | 81.5 | 45.1 | 67.9 | 36.5 |
|  | MP 68 | 68 | 1,00 | 19.3 | 340.0 | 3391 | 45.2 | 82.6 | 35.7 | 68.7 | 31.2 |
|  | MP 70 | 70 | 0,96 | 17.7 | 320.0 | 3059 | 33.4 | 81.9 | 26.4 | 68.4 | 22.9 |
|  | MP 72 | 72 | 0,82 | 16.1 | 320.0 | 2614 | 22.6 | 83.4 | 19.5 | 69.1 | 15.2 |
|  | MP 74 | 74 | 0,64 | 14.6 | 345.0 | 2207 | 13.4 | 81.5 | 11.1 | 67.2 | 8.8 |
|  | MP 76 | 76 | 0,79 | 13.0 | 369.0 | 2909 | 5.0 | 81.7 | 5.7 | 66.9 | 3.5 |
|  | MP 78 | 78 | 0,83 | 11.5 | 369.0 | 3062 | 2.4 | 80.5 | 1.9 | 68.7 | 1.6 |
|  | MP 80 | 80 | 0,91 | 10.1 | 369.0 | 3372 | 0.6 | 81.2 | 0.7 | 68.7 | 0.4 |
| **Sum/**  **Weighted means** | SI 50-69 | 59.8 | 12,14 | 26.9 | 286.1 | 33923 | 72.3 | 81.9 | 59.2 | 67.8 | 48.9 |
|  | SI 40-80 | 62.4 | 22,1 | 24.9 | 288.7 | 59312 | 57.8 | 81.9 | 47.5 | 67.9 | 39.1 |

a: two age groups combined in each case, b: The standard deviation is according to Poisson distribution the square root of n. BC, breast cancer cases, M, Million, MP, modeled populations, MS, mammography screening program, OS, overall survival, SI, Age interval for screening.

**Table S2:** Distribution of the breast cancer patient-relevant endpoints E1 to E6 in the modeled population cohorts with screening after 20 years of follow-up. Endpoints are defined as follows: E1=No event occurs during 20 years of follow-up, E2=Occurrence of tumor-independent deaths only, E3= Tumor-dependent deaths are always expected but in part prevented by tumor-independent deaths, E4=Tumor-related death is the first event to occur, E5= Tumor-independent death is the first event to occur prior to metastasis, E6=Tumor independent death occurs following diagnosis of metachronous metastasis. (E1+E2+E3 =100%, E3=E4+E5+E6).

|  |  | **Age [yrs]** | **BC [n]** | **E1 [%]** | **E2 [%]** | **E3 [%]** | **E4 [%]** | **E5 [%]** | **E6 [%]** | **LL** | **LL corr.^*^** | **YLL** | **YLL corr.^*^** |
| --- | --- | --- | --- | --- | --- | --- | --- | --- | --- | --- | --- | --- | --- |
| **Modeled screening cohort** | MC 40 | 40 | 1147 | 78.50 | 3.45 | 18.05 | 17.77 | 0.21 | 0.07 | 207 | 204 | 6900 | 6750 |
|  | MC 42 | 42 | 1392 | 77.55 | 4.27 | 18.18 | 17.87 | 0.22 | 0.10 | 253 | 249 | 7959 | 7770 |
|  | MC 44 | 44 | 1572 | 76.56 | 5.17 | 18.26 | 17.85 | 0.28 | 0.13 | 287 | 281 | 8520 | 8275 |
|  | MC 46 | 46 | 1780 | 75.49 | 6.17 | 18.34 | 17.81 | 0.40 | 0.13 | 326 | 317 | 9088 | 8777 |
|  | MC 48 | 48 | 2275 | 74.73 | 7.20 | 18.07 | 17.43 | 0.46 | 0.18 | 411 | 397 | 10740 | 10309 |
|  | MC 50 | 50 | 2909 | 73.19 | 8.70 | 18.11 | 17.28 | 0.62 | 0.21 | 527 | 503 | 12825 | 12191 |
|  | MC 52 | 52 | 3311 | 71.39 | 10.43 | 18.18 | 17.24 | 0.72 | 0.23 | 602 | 571 | 13606 | 12858 |
|  | MC 54 | 54 | 3292 | 69.52 | 12.35 | 18.13 | 16.99 | 0.85 | 0.29 | 597 | 559 | 12522 | 11714 |
|  | MC 56 | 56 | 3263 | 67.36 | 14.49 | 18.16 | 16.72 | 1.07 | 0.37 | 593 | 546 | 11458 | 10558 |
|  | MC 58 | 58 | 3388 | 65.01 | 16.91 | 18.08 | 16.48 | 1.20 | 0.40 | 613 | 558 | 10811 | 9882 |
|  | MC 60 | 60 | 3509 | 61.34 | 20.38 | 18.28 | 16.36 | 1.44 | 0.48 | 641 | 574 | 10321 | 9286 |
|  | MC 62 | 62 | 3558 | 56.89 | 24.90 | 18.20 | 15.96 | 1.66 | 0.58 | 648 | 568 | 9414 | 8342 |
|  | MC 64 | 64 | 3581 | 51.53 | 30.42 | 18.05 | 15.40 | 1.93 | 0.73 | 646 | 551 | 8429 | 7316 |
|  | MC 66 | 66 | 3721 | 44.14 | 37.57 | 18.28 | 14.97 | 2.44 | 0.88 | 680 | 557 | 7892 | 6653 |
|  | MC 68 | 68 | 3391 | 36.18 | 45.81 | 18.02 | 14.01 | 2.94 | 1.06 | 611 | 475 | 6279 | 5111 |
|  | MC 70 | 70 | 3059 | 27.24 | 54.65 | 18.10 | 13.24 | 3.59 | 1.28 | 554 | 405 | 4966 | 3884 |
|  | MC 72 | 72 | 2614 | 18.54 | 63.36 | 18.10 | 12.22 | 4.36 | 1.51 | 473 | 320 | 3712 | 2759 |
|  | MC 74 | 74 | 2207 | 10.60 | 71.11 | 18.29 | 10.97 | 5.56 | 1.77 | 404 | 242 | 2725 | 1875 |
|  | MC 76 | 76 | 2909 | 5.05 | 76.85 | 18.10 | 9.47 | 6.70 | 1.93 | 527 | 276 | 3056 | 1913 |
|  | MC 78 | 78 | 3062 | 1.89 | 79.90 | 18.21 | 8.13 | 8.07 | 2.02 | 558 | 249 | 2790 | 1560 |
|  | MC 80 | 80 | 3372 | 0.60 | 81.13 | 18.27 | 6.79 | 9.39 | 2.09 | 616 | 229 | 2632 | 1283 |
| **Sum/**  **Weighted means** | SI 50-69 | 59.8 | 33923 | 59.20 | 22.70 | 18.20 | 16.10 | 1.50 | 0.50 | 6158 | 5462 | 103557 | 93911 |

^*^, Adjusted for competing events E5 or E6. BC, breast cancer cases, LL, lives lost, YLL, years of life lost.

**Table S3:** Distribution of the breast cancer patient-relevant endpoints E1 to E6 in the modeled population cohorts without screening after 20 years of follow-up. Endpoints are defined as follows: E1=No event occurs during 20 years of follow-up, E2=Occurrence of tumor-independent deaths only, E3= Tumor-dependent deaths are always expected but in part prevented by tumor-independent deaths, E4=Tumor-related death is the first event to occur, E5= Tumor-independent death is the first event to occur prior to metastasis, E6=Tumor independent death occurs following diagnosis of metachronous metastasis. (E1+E2+E3 =100%, E3=E4+E5+E6).

|  |  | **Age [yrs]** | **BC [n]** | **E1 [%]** | **E2 [%]** | **E3 [%]** | **E4 [%]** | **E5 [%]** | **E6 [%]** | **LL** | **LL corr.^*^** | **YLL** | **YLL corr.^*^** |
| --- | --- | --- | --- | --- | --- | --- | --- | --- | --- | --- | --- | --- | --- |
| **Modeled control cohort** | MC 40 | 40 | 1147 | 64.74 | 2.91 | 32.35 | 31.86 | 0.35 | 0.14 | 371 | 365 | 12578 | 12318 |
|  | MC 42 | 42 | 1392 | 64.32 | 3.57 | 32.11 | 31.53 | 0.43 | 0.15 | 447 | 439 | 14291 | 13956 |
|  | MC 44 | 44 | 1572 | 63.51 | 4.30 | 32.19 | 31.44 | 0.54 | 0.22 | 506 | 494 | 15295 | 14858 |
|  | MC 46 | 46 | 1780 | 62.66 | 5.04 | 32.30 | 31.37 | 0.67 | 0.26 | 575 | 558 | 16324 | 15781 |
|  | MC 48 | 48 | 2275 | 61.66 | 6.10 | 32.24 | 31.13 | 0.82 | 0.29 | 733 | 708 | 19524 | 18780 |
|  | MC 50 | 50 | 2909 | 60.86 | 7.23 | 31.90 | 30.62 | 0.95 | 0.34 | 928 | 890 | 23079 | 22062 |
|  | MC 52 | 52 | 3311 | 59.40 | 8.52 | 32.08 | 30.48 | 1.18 | 0.42 | 1062 | 1009 | 24560 | 23280 |
|  | MC 54 | 54 | 3292 | 57.69 | 10.18 | 32.13 | 30.28 | 1.39 | 0.45 | 1058 | 997 | 22683 | 21349 |
|  | MC 56 | 56 | 3263 | 55.67 | 12.00 | 32.34 | 30.07 | 1.68 | 0.58 | 1055 | 981 | 20891 | 19439 |
|  | MC 58 | 58 | 3388 | 53.82 | 14.12 | 32.06 | 29.49 | 1.88 | 0.69 | 1086 | 999 | 19745 | 18213 |
|  | MC 60 | 60 | 3509 | 51.18 | 17.02 | 31.79 | 28.67 | 2.34 | 0.78 | 1116 | 1006 | 18475 | 16780 |
|  | MC 62 | 62 | 3558 | 47.27 | 20.62 | 32.11 | 28.45 | 2.68 | 0.98 | 1142 | 1012 | 17175 | 15382 |
|  | MC 64 | 64 | 3581 | 42.63 | 25.21 | 32.16 | 27.70 | 3.34 | 1.13 | 1152 | 992 | 15560 | 13667 |
|  | MC 66 | 66 | 3721 | 36.91 | 31.18 | 31.91 | 26.54 | 3.90 | 1.47 | 1187 | 988 | 14350 | 12302 |
|  | MC 68 | 68 | 3391 | 29.96 | 37.92 | 32.12 | 25.47 | 4.86 | 1.79 | 1089 | 864 | 11621 | 9654 |
|  | MC 70 | 70 | 3059 | 22.87 | 45.31 | 31.82 | 23.70 | 5.86 | 2.25 | 973 | 725 | 9134 | 7291 |
|  | MC 72 | 72 | 2614 | 15.42 | 52.64 | 31.94 | 22.13 | 7.23 | 2.57 | 835 | 579 | 6845 | 5220 |
|  | MC 74 | 74 | 2207 | 8.83 | 59.21 | 31.96 | 20.09 | 8.94 | 2.94 | 705 | 443 | 4998 | 3586 |
|  | MC 76 | 76 | 2909 | 4.15 | 63.87 | 31.98 | 17.80 | 10.85 | 3.33 | 930 | 518 | 5692 | 3766 |
|  | MC 78 | 78 | 3062 | 1.55 | 66.32 | 32.13 | 15.35 | 13.29 | 3.49 | 984 | 470 | 5178 | 3087 |
|  | MC 80 | 80 | 3372 | 0.47 | 67.46 | 32.07 | 12.90 | 15.54 | 3.63 | 1081 | 435 | 4870 | 2562 |
| **Sum/**  **Weighted means** | SI 50-69 | 59.8 | 33923 | 49.10 | 18.80 | 32.10 | 28.70 | 2.50 | 0.90 | 10875 | 9738 | 188139 | 172128 |

^*^, Adjusted for competing events E5 or E6. BC, breast cancer cases, LL, lives lost, YLL, years of life lost.

**Table S4:** Screening effects comparing modeled cohorts with (MS+) and without screening (MS-) at 20 years of follow-up. Cost per screening examination € 88.

|  |  | **PD** | **PD corr.*** | **MSE per PD corr.*** | **€ per PD corr.*** | **LY corr.*** | **MSE per LY corr.*** | **€ per LY corr.*** | **PD per 10^4^ WS** | **LY per 10^4^WS** |
| --- | --- | --- | --- | --- | --- | --- | --- | --- | --- | --- |
| **∆+MS/-MS (Tab.2/3)** | ∆40 | 164 | 161 | 3208 | 282000 | 5678 | 93 | 8200 | 3.1 | 108 |
|  | ∆42 | 194 | 190 | 2599 | 229000 | 6332 | 80 | 7000 | 3.8 | 125 |
|  | ∆44 | 219 | 213 | 2236 | 197000 | 6775 | 72 | 6400 | 4.5 | 138 |
|  | ∆46 | 249 | 241 | 1985 | 175000 | 7236 | 68 | 6000 | 5.0 | 146 |
|  | ∆48 | 323 | 311 | 1742 | 153000 | 8784 | 64 | 5600 | 5.7 | 156 |
|  | ∆50 | 401 | 387 | 1566 | 138000 | 10253 | 61 | 5400 | 6.4 | 163 |
|  | ∆52 | 460 | 438 | 1512 | 133000 | 10954 | 64 | 5600 | 6.6 | 157 |
|  | ∆54 | 461 | 438 | 1566 | 138000 | 10162 | 71 | 6300 | 6.4 | 140 |
|  | ∆56 | 463 | 435 | 1596 | 140000 | 9433 | 78 | 6900 | 6.3 | 128 |
|  | ∆58 | 474 | 441 | 1507 | 133000 | 8934 | 80 | 7000 | 6.6 | 125 |
|  | ∆60 | 475 | 432 | 1450 | 128000 | 8154 | 84 | 7400 | 6.9 | 120 |
|  | ∆62 | 494 | 444 | 1292 | 114000 | 7761 | 82 | 7200 | 7.7 | 123 |
|  | ∆64 | 505 | 441 | 1230 | 108000 | 7130 | 85 | 7500 | 8.1 | 117 |
|  | ∆66 | 507 | 431 | 1199 | 106000 | 6458 | 91 | 8100 | 8.3 | 109 |
|  | ∆68 | 478 | 389 | 1282 | 113000 | 5341 | 110 | 9700 | 7.8 | 91 |
|  | ∆70 | 419 | 320 | 1494 | 131000 | 4168 | 140 | 12300 | 6.7 | 71 |
|  | ∆72 | 362 | 259 | 1577 | 139000 | 3133 | 166 | 14600 | 6.3 | 60 |
|  | ∆74 | 301 | 201 | 1591 | 140000 | 2273 | 187 | 16500 | 6.3 | 53 |
|  | ∆76 | 404 | 242 | 1629 | 143000 | 2637 | 213 | 18700 | 6.1 | 47 |
|  | ∆78 | 427 | 221 | 1877 | 165000 | 2388 | 272 | 23900 | 5.3 | 37 |
|  | ∆80 | 465 | 206 | 2218 | 195000 | 2238 | 357 | 31400 | 4.5 | 28 |
| **Sum/**  **Weighted means for 10 Studies** | SI 50-69 | 4718 | 4276 | 1413 | 124000 | 8387 | 81 | 7200 | 7.1 | 126 |

*, Adjusted for competing events, LY, life years gained, MS, mammography screening program, MSE, mammography screening examinations, PD, prevented deaths,

SI, screening interval, WS, Women screened.

**Table S5:** Equivalent results compared to above for the United States of America using data obtained from the SEER database. Cost per screening examination $70.

|  |  | **Age^a^ [yrs]** | **Population**  **[M]** | **Incidence per 10^5^** | **BC [n]** | **PD** | | **PD corr.*** | | **MSE per PD corr.*** | | **$ per PD corr.*** | | **LY corr.*** | | **MSE per LY corr.*** | | **$ per LY corr.*** | | **PD per 10^4^ WS** | | **LY per 10^4^WS** | |
| --- | --- | --- | --- | --- | --- | --- | --- | --- | --- | --- | --- | --- | --- | --- | --- | --- | --- | --- | --- | --- | --- | --- | --- |
| **∆+MS/-MS** | ∆40 | 40 | 4.2 | 111 | 4666 | 654 | 645 | | 3259 | | 287000 | | 22625 | | 95 | | 8.3 | | 3.1 | | 106 | |  |
|  | ∆42 | 42 | 4.2 | 141 | 5928 | 830 | 814 | | 2582 | | 227000 | | 27126 | | 79 | | 7 | | 3.9 | | 126 | |  |
|  | ∆44 | 44 | 3.96 | 165 | 6541 | 913 | 895 | | 2215 | | 195000 | | 28276 | | 72 | | 6.3 | | 4.5 | | 139 | |  |
|  | ∆46 | 46 | 3.96 | 186 | 7373 | 1021 | 997 | | 1988 | | 175000 | | 29676 | | 69 | | 6 | | 5 | | 146 | |  |
|  | ∆48 | 48 | 4.08 | 210 | 8560 | 1173 | 1137 | | 1792 | | 158000 | | 32110 | | 66 | | 5.8 | | 5.6 | | 152 | |  |
|  | ∆50 | 50 | 4.19 | 240 | 10051 | 1398 | 1348 | | 1553 | | 137000 | | 35737 | | 61 | | 5.4 | | 6.4 | | 164 | |  |
|  | ∆52 | 52 | 4.19 | 250 | 10470 | 1445 | 1382 | | 1515 | | 133000 | | 34451 | | 64 | | 5.6 | | 6.6 | | 157 | |  |
|  | ∆54 | 54 | 4.38 | 260 | 11378 | 1578 | 1491 | | 1467 | | 129000 | | 34917 | | 66 | | 5.8 | | 6.8 | | 151 | |  |
|  | ∆56 | 56 | 4.38 | 270 | 11815 | 1645 | 1540 | | 1421 | | 125000 | | 33730 | | 69 | | 6.1 | | 7 | | 145 | |  |
|  | ∆58 | 58 | 4.37 | 290 | 12667 | 1762 | 1636 | | 1335 | | 117000 | | 33193 | | 71 | | 6.2 | | 7.5 | | 142 | |  |
|  | ∆60 | 60 | 4.36 | 325 | 14170 | 1961 | 1792 | | 1217 | | 107000 | | 33726 | | 70 | | 6.2 | | 8.2 | | 142 | |  |
|  | ∆62 | 62 | 4.36 | 360 | 15696 | 2188 | 1954 | | 1116 | | 98000 | | 34140 | | 71 | | 6.2 | | 9 | | 142 | |  |
|  | ∆64 | 64 | 3.86 | 390 | 15054 | 2087 | 1829 | | 1055 | | 93000 | | 29451 | | 73 | | 6.5 | | 9.5 | | 136 | |  |
|  | ∆66 | 66 | 3.86 | 425 | 16405 | 2244 | 1898 | | 1017 | | 89000 | | 28446 | | 78 | | 6.9 | | 9.8 | | 128 | |  |
|  | ∆68 | 68 | 3.56 | 445 | 15842 | 2214 | 1798 | | 990 | | 87000 | | 24916 | | 84 | | 7.4 | | 10.1 | | 119 | |  |
|  | ∆70 | 70 | 3.26 | 460 | 14996 | 2062 | 1601 | | 1018 | | 90000 | | 20501 | | 96 | | 8.5 | | 9.8 | | 104 | |  |
|  | ∆72 | 72 | 3.26 | 468 | 15257 | 2116 | 1528 | | 1067 | | 94000 | | 18393 | | 112 | | 9.9 | | 9.4 | | 89 | |  |
|  | ∆74 | 74 | 2.17 | 464 | 10078 | 1392 | 926 | | 1173 | | 103000 | | 10538 | | 137 | | 12 | | 8.5 | | 73 | |  |
|  | ∆76 | 76 | 2.17 | 460 | 9991 | 1388 | 831 | | 1307 | | 115000 | | 9056 | | 171 | | 15 | | 7.7 | | 59 | |  |
|  | ∆78 | 78 | 1.81 | 450 | 8136 | 1126 | 586 | | 1543 | | 136000 | | 6320 | | 224 | | 19.7 | | 6.5 | | 45 | |  |
|  | ∆80 | 80 | 1.44 | 437 | 6310 | 873 | 396 | | 1823 | | 160000 | | 4191 | | 296 | | 26.1 | | 5.5 | | 34 | |  |
| **Sum/**  **Weighted means for 10 Studies** | SI 50-69 | 60.4 | 41.5 | 337 | 133548 | 18522 | 16668 | | 1235 | | 108000 | | 31859 | | 72 | | 6.3 | | 8.3 | | 141 | |  |

*, Adjusted for competing events, LY, life years gained, MS, mammography screening, PD, prevented deaths,

MSE, screening examinations, SI, screening interval, WS, Women screened.

**Figure S1:** 20-year OS of a healthy cohort population, OS of patients with BC, and BC-specific survival of the age cohorts 60-61 with (+MS) and without screening (-MS). These cohorts are equivalent to the expected prognosis of all BCs occurring between ages 50 and 70 in a population of 12.1 million women.


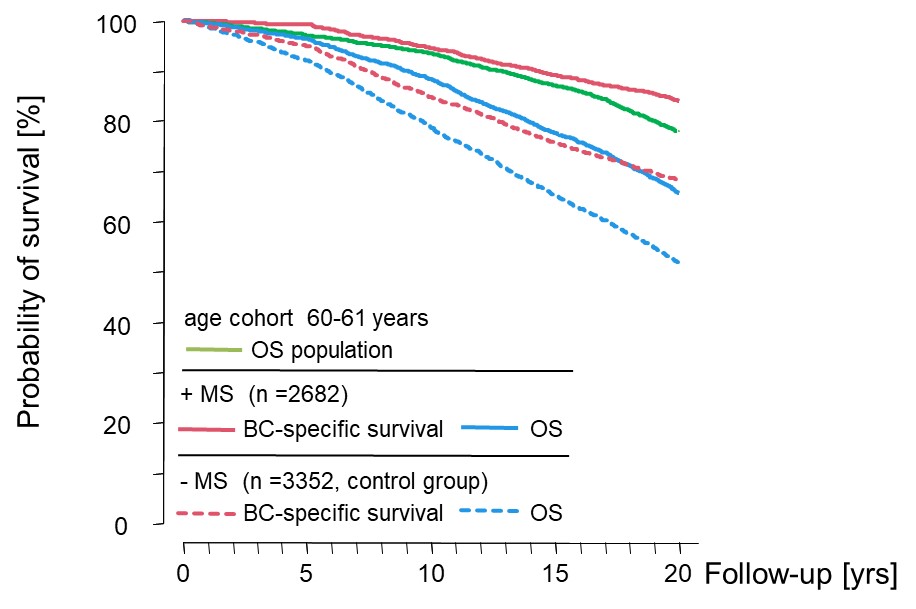


**Figure S2:** 20-year OS of a healthy cohort population, OS of patients with BC, BC-specific survival of age cohorts 40-41 years with (+MS) and without screening (-MS).


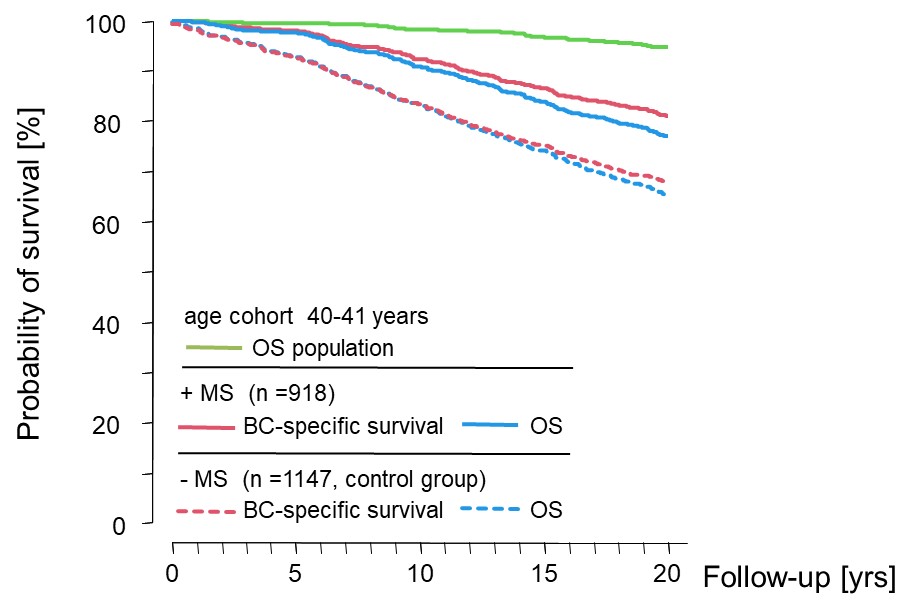


The validity of the modeling results is also illustrated by the adjustment to the UK Age trial*1. The survival curves above are one realization of the modeling of study data with a screening and control group ages 40-41 and 20 years of follow-up. (Table 2: MS 40, Table 3: MC 40).

The accumulation of the studies from 40 to 48 years provides the results of the cohorts MP 40 after 10 years:

Table 1:

**+MS**

n= 7 126 cases of BC

**-MS**

n=5 701 cases of BC and 5 010 000 person-years

The corresponding results of the UK Age trial are:

**+MS**

n=835 BCs

n=569 632 person-years

**-MS**

n=1628 BCs

n=1 129 985 person-years

The results of the UK trial with the corresponding person-years are in +MS/-MS 810/1607 compared to 835/1628. The 10-year survival for +MS/-MS is 92.0%/82.8% with 73/181 BC-related death compared to 83/219 BC death. The relative rates of +MS/-MS are after 10 years 0.46 and 0.65 after 20 years compared to RR: 0.75/0.98 The modeled survival of both groups is consistent with population-based outcomes for our region of 4.9 million.

The current 5- and 10-year survival used results in a slightly better prognosis after 20 years and partly explains the slightly higher mortality despite the favorable prognostic factors in the +MS- cohort of the +MS cohort. *3 Age-specific analyses including information on molecular subtypes from screening, registry, and clinical studies can further increase the power of modeling, especially the evaluation of extended age intervals for screening (SI).

*1 S. W. Duffy, D. Vulkan, H. Cuckle, D. Parmar, S. Sheikh, R. A. Smith, et al. Effect of mammographic screening from age 40 years on breast cancer mortality (UK Age trial): final results of a randomised, controlled trial. Lancet Oncol 2020 Vol. 21 Issue 9 Pages 1165-1172

*2 Tumorregister München: http://www.tumorregister-muenchen.de/facts/specific_analysis.php (updated May 2022)

*3 S. Duffy, D. Vulkan, H. Cuckle, D. Parmar, S. Sheikh, R. Smith, et al. Annual mammographic screening to reduce breast cancer mortality in women from age 40 years: long-term follow-up of the UK Age RCT. Health Technol Assess 2020 Vol. 24 Issue 55 Pages 1-24
